# iAMP-SeE: an antimicrobial peptide recognition model based on ESM2 feature extraction and hybrid attention mechanisms

**DOI:** 10.7717/peerj.20978

**Published:** 2026-03-26

**Authors:** QingWei Chen, ShuMei Li, Liang Huang, XiangYu Yu, Dan Xu, Zhao Qi

**Affiliations:** 1 School of Information and Artificial Intelligence, Anhui Agricultural University, Hefei, Anhui Province, China; 2 Anhui Province Key Laboratory of Smart Agricultural Technology and Equipment, Hefei, Anhui Province, China

**Keywords:** Antimicrobial peptide prediction, Deep learning, CNN, BiLSTM, SE, ECA

## Abstract

**Background:**

Antimicrobial peptides (AMPs) are short peptides with diverse biological activities and playing a crucial role in various biological processes. Due to the widespread misuse of traditional antibiotics and the increasing resistance of microorganisms to these drugs, AMPs have emerged as a promising alternative. Consequently, the identification of AMPs has garnered significant research interest. Numerous computational methods based on machine learning algorithms have been developed to facilitate AMP recognition. However, some existing AMPs recognition models only focus on binary classification tasks or only identify the functional activity of a limited number of AMPs categories in multi-class classification tasks. To address this limitation, this study proposes a two-stage AMPs recognition model, iAMP-SeE.

**Methods:**

The iAMP-SeE model extracts features from protein sequences using ESM2, employs a Convolutional Neural Network (CNN) module to capture local patterns from ESM features and utilizes a Bidirectional Long Short-Term Memory (BiLSTM) network to capture long-term dependencies. Furthermore, it incorporates Squeeze-and-Excitation (SE) and Efficient Channel Attention (ECA) mechanisms, which focus on global and local channel relationships, respectively. These two attention mechanisms are complementary, as they enhance features across various dimensions and granularities while simultaneously suppressing irrelevant or redundant features, thereby boosting the model’s performance. Additionally, to address the issue of imbalanced datasets, the Synthetic Minority Over-sampling Technique (SMOTE) is incorporated into the multi-classification task. This method balances the number of AMP categories and ensures that minority classes are not overlooked during model training.

**Results:**

Evaluation across a range of classification thresholds demonstrated the stability of the model’s performance metrics in both binary and multi-class tasks. Furthermore, comparative experiments with existing AMP recognition models confirmed the superior performance of iAMP-SeE.

**Conclusions:**

Rigorous experimental comparisons and ablation studies demonstrate the effectiveness of iAMP-SeE for both binary and multi-class AMP classification tasks. The source code is publicly available at: https://github.com/cqw0715/iAMP-SeE.git.

## Introduction

Antimicrobial peptides (AMPs) are a class of short bioactive peptides produced by the innate immune system of organisms (Ageitos et al., 2016). They are widely present in humans, animals, plants, and even microorganisms (Peng et al., 2023). AMPs exert potent antimicrobial effects primarily by directly disrupting the integrity of pathogen cell membranes (Zhao et al., 2025b), interfering with cellular metabolism (Elbediwi & Rolff, 2024), and modulating host immune responses (Fusco et al., 2022). This unique multi-target mechanism endows AMPs with significant inhibitory activity against Gram-positive bacteria (Müller et al., 2025), Gram-negative bacteria (Xiao et al., 2025), fungi (Damica et al., 2025), viruses (Yang et al., 2025), and even cancer cells (Setiawan, Rudan & Wolf, 2025). Based on these advantages, AMPs are considered to be the most promising new antimicrobial agents to replace traditional antibiotics, representing an important direction for future antimicrobial drug research and development (Peggion et al., 2025). Currently, AMPs have demonstrated significant application value in various fields, such as the treatment of drug-resistant bacterial infections (Alisigwe, Ikpa & Otuonye, 2025), sepsis prevention (Ibrahim et al., 2024), improving livestock production, and food preservation (Shao et al., 2024).

While traditional experimental methods for AMP recognition are reliable, they are subject to several limitations, including low throughput, high cost, lengthy duration, and the time-consuming process of handling only a small number of samples at a time (Zhang et al., 2023). With the rapid advancement of bioinformatics and deep learning technologies, Convolutional Neural Network (CNN), Transformer, and other related models, leveraging their capacity to automatically learn sequence features, can efficiently capture the essential characteristics of AMPs, facilitating their identification and classification. This has transformed the use of computational models for AMP recognition into a significant research hotspot.

In the field of AMP recognition and prediction, extensive research has been dedicated to binary classification tasks. For instance, Wang et al. (2023) extracted relevant information from protein sequences using the Pseudo Amino Acid Composition (PseAAC) feature extraction method, employed a three-layer Bidirectional Long Short-Term Memory (BiLSTM) combined with relevant baseline machine learning models such as support vector machine (SVM) and K-nearest neighbor (KNN), and utilized Adaptive Boosting (AdaBoost), extreme gradient boosting (XGBoost), and other integrated learning methods in experimental testing. Ultimately, the AMP-EBiLSTM model achieved a test accuracy of 92.39% on its self-built dataset, and ablation studies demonstrated that the BiLSTM module significantly enhanced the model’s classification performance. Xing et al. (2023) were the first to combine BERT (Bidirectional Encoder Representations from Transformers) feature extraction with the CNN-BiLSTM-Attention-based iAMP-Attenpred deep learning model. Through 10-fold cross validation, they confirmed that the model achieved an accuracy rate of 98.44% on its dataset, demonstrating that the combined CNN-BiLSTM-Attention model can efficiently extract relevant information from antimicrobial peptide sequences for binary classification tasks. In the multi-classification task of AMPs, numerous studies have garnered significant attention. For instance, Jing and his team developed the iAMPCN model for AMPs recognition, which is applicable to both binary and multi-classification tasks. This model employs a CNN structure to extract features, utilizes various filters to capture features at different scales, and incorporates a transfer learning strategy for the recognition and classification of AMPs (Jing et al., 2023). Zhao et al. (2025a) employed the ESM-2 model for sequence feature extraction, integrating CNN, BiLSTM, and CBAM (Convolutional Block Attention Model) attention mechanism to propose the deep-AMPpred model for AMPs binary and multi-classification tasks. CNN was utilized to capture local features, BiLSTM was employed to model long-term and short-term dependencies, and the CBAM was used to emphasize important features. The excellent performance, maintaining an AUC of 0.9642 on the independent test set, and good generalization ability were proven through 5-fold cross-validation.

The aforementioned studies have demonstrated that integrating CNN, BiLSTM, and an adaptive attention mechanism enables models to effectively capture recognize pertinent information within AMP protein sequences and perform classification. Nevertheless, several issues remain unresolved in these studies. First, some of the aforementioned studies focus solely on either binary or multi-class classification tasks for AMPs, and their proposed deep learning models cannot simultaneously address both types of classification tasks or perform poorly in the other scenario. Second, in the context of AMP binary classification, several studies have not adequately addressed the impact of class imbalance between positive and negative samples on model outcomes. Significant disparity in the number of samples from either class may lead the model to exhibit prediction bias toward the majority class, thereby causing it to overlook biologically relevant sequence information and fail to reflect the true model performance. Finally, in multi-class classification tasks, severe imbalance in the number of samples across different functional categories of AMPs can prevent the model from accurately distinguishing between them. This may result in overfitting to the majority classes or underfitting to the minority classes, ultimately leading to unreliable predictions.

To address these issues, this study developed iAMP-SeE, a model for binary and multi-class classification of AMPs. This model uses the ESM2 method to extract features from AMP protein sequences. It then employs a 1D-CNN convolution kernel to scan the input sequence, capturing local patterns within the amino acid sequences. A BiLSTM network is used to capture global dependencies through its bidirectional structure. By integrating Squeeze-and-Excitation (SE) (Nguyen & Kang, 2025) and Efficient Channel Attention (ECA) (Li et al., 2025) attention mechanisms, the model uses a multi-granularity and complementary feature enhancement strategy to improve its ability to capture key information from AMP protein sequences.

This study constructed a dataset with balanced positive and negative samples and a similar range of sequence lengths for binary classification of AMPs. For the multi-class task, the Synthetic Minority Oversampling Technique (SMOTE) method (Chhillar & Singh, 2025) was employed to address class imbalance by generating synthetic samples for minority classes through linear interpolation, increasing their quantity to match that of the majority class. By evaluating the classification performance of models under different dataset identity thresholds in both binary and multi-class tasks, assessing their generalization capabilities with both balanced and imbalanced positive/negative sample distributions, and comparing them with existing AMPs recognition models, the superior performance of the iAMP-SeE model has been validated.

## Materials & Methods

The hardware configuration employed in this study comprises an Intel(R) Xeon(R) E5-2682 v4 CPU and an NVIDIA GeForce RTX 3060-12G GPU. The system operates on the Linux kernel version 6.14, with the following relevant software versions: Python 3.10, TensorFlow 2.13.0, PyTorch 2.9.1+cu12, and fair-esm 2.0.0.

**Figure 1:**
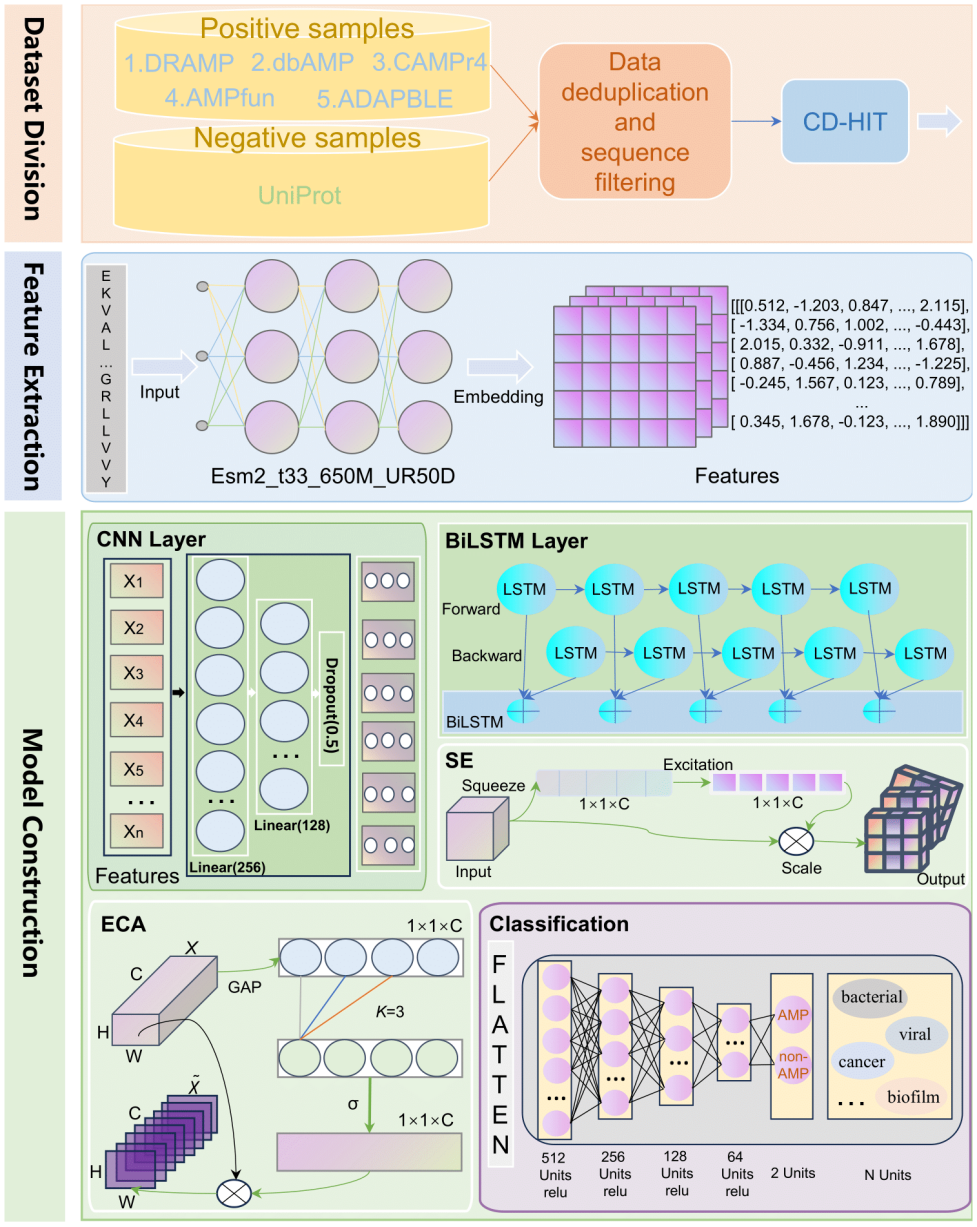
Overall architecture of iAMP-SeE.

To facilitate a better understanding of the iAMP-SeE model, this paper elaborates on its data collection, feature extraction, and the construction of its core components. We have also included a flowchart (Fig. 1) to visually represent each module of the model. The specific details of the flowchart will be further explained in subsequent chapters.

### Datasets

**Figure 2:**
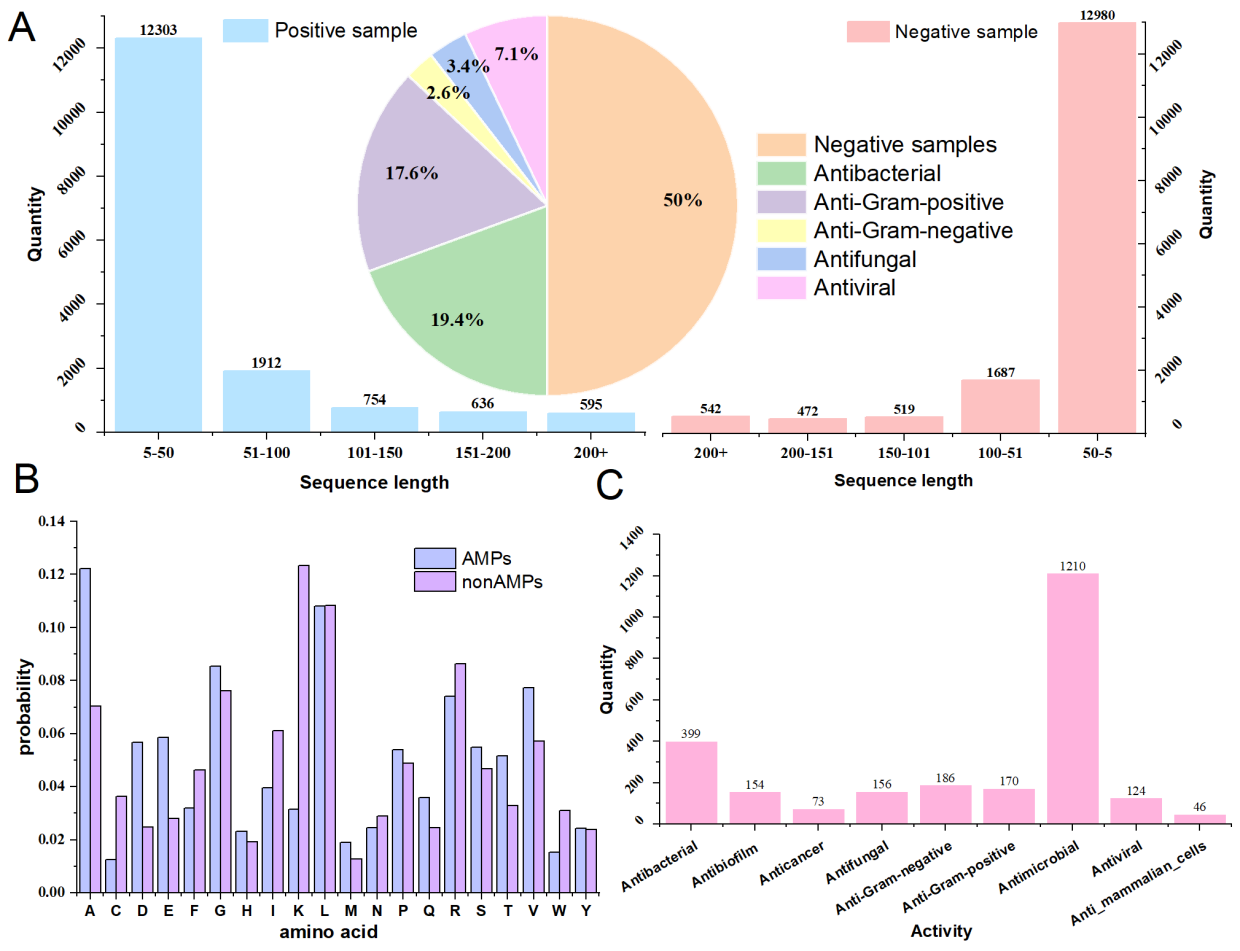
Dataset description diagram.

In this study, we utilized two datasets. Dataset 1 contains both AMPs and non-AMPs. It includes 16,200 unique positive sequences, which are antimicrobial peptides obtained from five public databases: DRAMP (Ma et al., 2025), dbAMP (Jhong et al., 2021), CAMPr-4 (Gawde et al., 2022), AMPfun (Chung et al., 2019), and ADAPTABLE (Ramos-Martín et al., 2019). These positive sequences are categorized into five categories: ‘Antibacterial’, ‘Anti-Gram-positive’, ‘Anti-Gram-negative’, ‘Antifungal’, and ‘Antiviral’. The negative samples, which are non-AMPs, were collected from the UniProt database. We first excluded any protein sequences annotated with the five AMP categories mentioned above. Then, we screened for relevant sequences based on the length distribution of the positive samples and used the CD-HIT tool (Fu et al., 2012) with a 100% threshold to remove duplicate sequences. This process yielded 16,200 negative sequences. Dataset 1 is designed to have an equal number of positive and negative samples with similar sequence length ranges. This balance prevents the model from being biased toward either class during training due to large disparities in sample quantity or length. The detailed classification and sequence length distribution of Dataset 1 are shown in Fig. 2A, while the amino acid composition of both positive and negative samples is displayed in Fig. 2B.

Dataset 2 originates from the dataset utilized by Zhao et al. (2024) in the deep-AMPpred model for the AMPs multi-classification task. This dataset comprises nine different categories of antimicrobial peptides, as detailed in Fig. 2C. Compared to Dataset 1, Dataset 2 includes a broader range of AMP categories. We employed this dataset to enable a comprehensive comparison of iAMP-SeE’s performance metrics with other models in our multi-class classification tasks.

### Feature extraction

We conducted a comprehensive evaluation of four traditional protein feature extraction methods—Amino Acid Composition (AAC), Dipeptide Composition (DPC), Word2Vec word embedding, and Positional Encoding—as well as two novel techniques, ESM2 and ProteinBERT. A detailed introduction to each of these methods is provided below.

### AAC

AAC is a fundamental feature extraction technique in protein sequence analysis. It characterizes a protein sequence by calculating the frequency of each amino acid within that sequence (Nakashima, Nishikawa & Ooi, 2008). The formula for this calculation is:

(1) \begin{eqnarray*}\mathrm{AAC}= \frac{N}{L} .\end{eqnarray*}

In this context, N stands for the number of occurrences of amino acids in the sequence, while L denotes the total length of the sequence. Upon undergoing AAC feature extraction, the sequence will produce a 20-dimensional feature vector.

### DPC

DPC is a feature extraction method for protein sequences. It calculates the frequency of all adjacent amino acid pairs in a sequence, converting the protein sequence into a 400-dimensional fixed-length feature vector (Saravanan & Gautham, 2015). The mathematical formula is as follows:

(2) \begin{eqnarray*}\mathrm{DPC}= \frac{N}{L-1} .\end{eqnarray*}

N represents the number of dipeptides that appear in the sequence, counted using a sliding window, while L-1 denotes the total number of possible dipeptides in the sequence. When L ≤ 1, to avoid division by zero errors, the default setting is total = 1.

### Word2Vec

Word2Vec is a neural network-based word embedding technique that originated in natural language processing (NLP). It has since been successfully applied to bioinformatics for sequence analysis. By training a shallow neural network, Word2Vec maps discrete symbols into a continuous vector space, where symbols with similar functions or semantics are placed in close proximity (Mikolov et al., 2013). The relevant formula is as follows:

(3) \begin{eqnarray*}Word2Vec=-\log \nolimits \sigma \left( {\mathbf{u}}_{c}^{\top }{\mathbf{v}}_{w} \right) -\sum _{k=1}^{K}\log \nolimits \sigma \left( -{\mathbf{u}}_{k}^{\top }{\mathbf{v}}_{w} \right) .\end{eqnarray*}

In the aforementioned formula, *Vw* represents the central word vector, *u*${}_{c}^{\top }$ stands for the correct context word vector, *u*${}_{k}^{\top }$ denotes the incorrect word vector obtained through negative sampling, *K* signifies the number of negative samples, and *σ* represents the sigmoid function. Following Word2Vec feature extraction, the model yields a 200-dimensional feature vector. Word2Vec has emerged as a pivotal method for biological sequence feature extraction, frequently employed in conjunction with other features to enhance prediction performance.

### Positional Encoding

Positional Encoding is a technique used to add positional information to sequence data. Since the Transformer’s self-attention mechanism doesn’t inherently contain sequence order, Positional Encoding uses sine and cosine functions to generate positional encodings. These encodings are then combined with the original input embeddings, giving each element a unique code based on its position. This enables the model to differentiate between elements at various locations in the sequence (Petersen & Posner, 2012). The calculation formula for Positional Encoding is as follows:

(4) \begin{eqnarray*} \left\{ \begin{array}{@{}l@{}} \displaystyle P{E}_{(pos,2i)}=\sin \nolimits \left( \frac{pos}{1000{0}^{2i/{d}_{\mathrm{model}}}} \right) \\ \displaystyle P{E}_{(pos,2i+1)}=\cos \nolimits \left( \frac{pos}{1000{0}^{2i/{d}_{\mathrm{model}}}} \right) \end{array} \right. \end{eqnarray*}

where *pos* represents the position of the word in the sequence, *d*_*model*_ denotes the dimension of the word vector, and *i* represents the current dimension index. After positional encoding feature extraction, a 300-dimensional feature vector can be obtained.

### ESM2

ESM2 extracts protein sequence features using a Transformer architecture. The process begins by converting the input sequence into tokens and assigning them positional embeddings. After 33 layers of self-attention computation, the hidden state for each amino acid position is extracted from the final layer (Mall et al., 2024). This results in an output feature vector with a dimension of 1,280. The full sequence is represented as a tensor of (sequence length, 1,280). A flowchart of this process is shown in Fig. 1.

### ProteinBERT

ProteinBERT is a protein language model based that uses the BERT architecture. It learns biological features from large-scale protein sequences through a self-supervised pre-training process (Hu, Kuang & Yang, 2025). To extract features, the input sequence is segmented into amino acid tokens with positional encodings added. After processing through a 12-layer Transformer encoder with bidirectional attention, features are extracted from either the [CLS] special token or the hidden state of the final layer. The model’s output has a dimension of 1,024 and can generate either global features for the entire sequence or local features for individual amino acids, making it suitable for functional prediction and structural analysis.

### Performance evaluation

In this study, five evaluation metrics are selected to assess the classification performance of the proposed method. The calculation formulas for these metrics are as follows:

(5) \begin{eqnarray*} \left\{ \begin{array}{@{}l@{}} \displaystyle ACC= \frac{TP+TN}{TP+TN+FP+FN} \\ \displaystyle SEN= \frac{TP}{TP+FN} \\ \displaystyle SPE= \frac{TN}{TN+FP} \\ \displaystyle MCC= \frac{(TP\times TN)-(FP\times FN)}{\sqrt{(TP+FP)(TP+FN)(TN+FP)(TN+FN)}} \\ \displaystyle F1\mathrm{_}score= \frac{2\times TP}{2\times TP+FP+FN} . \end{array} \right. \end{eqnarray*}

In the context of the metrics mentioned, TP, FP, TN, and FN stand for true positives, false positives, true negatives, and false negatives, respectively. Accuracy (ACC) measures the model’s overall prediction accuracy, while Matthews Correlation Coefficient (MCC) reflects the Pearson correlation between predicted and actual classifications. Sensitivity (SEN) gauges the model’s ability to detect true positives, and Specificity (SPE) evaluates its capacity to correctly identify true negatives. The F1-score balances the comprehensive performance of precision and recall. These five metrics are commonly used to evaluate the performance of statistical models. Additionally, to assess the model’s true positive rate (TPR) and false positive rate (FPR) across different thresholds and to evaluate its discriminative ability, we use the Area Under the Curve (AUC) of the Receiver Operating Characteristic (ROC) curve. The core formula is as follows:

(6) \begin{eqnarray*} \left\{ \begin{array}{@{}l@{}} \displaystyle FPR= \frac{FP}{FP+TN} \vskip{2pt}\\ \displaystyle TPR= \frac{TP}{TP+FN} . \end{array} \right. \end{eqnarray*}

### Model building

#### CNN

The CNN layer consists of two one-dimensional convolutional layers (Conv1D). The first layer uses 256 filters with a kernel size of 5, while the second layer has 128 filters with a kernel size of 3. Both layers use “same” padding and the Swish activation function. Each layer is followed by Batch Normalization to stabilize training and a 50% Dropout to prevent overfitting. The operation of the CNN layer is represented by the following formula:

(7) \begin{eqnarray*}H=\mathrm{Dropout}(\mathrm{BN}(\mathrm{swish}({W}_{2}\times \mathrm{BN}(\mathrm{swish}({W}_{1}\times X+{b}_{1}))+{b}_{2}))).\end{eqnarray*}

In the formula, *H* is the final output of the CNN layer, BN stands for batch normalization, which standardizes the output of each layer. *b*_1_ and *b*_2_ are the bias terms of the convolutional layer, *W*_1_ and *W*_2_ represent the weight matrices of the convolutional layer, and X denotes the input feature. These two CNN layers extract hierarchical sequential patterns ranging from broad scope to local details from the input ESM2 features by gradually reducing the kernel size and the number of channels, providing a local feature foundation for the subsequent BiLSTM layer and attention mechanism.

#### BiLSTM

BiLSTM is a recurrent neural network that processes sequences in both forward and backward directions to effectively capture long-term dependencies. This dual-directional approach generates a more comprehensive and detailed feature representation, which is ideal for sequential data. In this study, we used a two-layer BiLSTM network for hierarchical feature extraction. The first layer has 256 units to capture basic sequence patterns, while the second layer has 128 units for extracting higher-order features. The output from the BiLSTM network is then passed to the attention mechanism layer.

#### Attention mechanism

In deep learning models, attention mechanisms dynamically adjust feature importance to improve overall performance. To enhance our model’s ability to recognize AMPs in protein sequences, we integrated two efficient attention modules: SE and ECA.

SE is a lightweight channel attention mechanism. It first compresses spatial information and captures global channel features using global average pooling. It then uses a fully connected layer to learn inter-channel dependencies and generate weights. Finally, it enhances important features and suppresses irrelevant ones through channel weighting. The goal is to boost the feature representation of CNN networks and, in turn, the overall model performance. The relevant formulas are as follows:

(8) \begin{eqnarray*}{\mathrm{Output}}_{\mathrm{SE}}=\sigma ({W}_{2}\delta ({W}_{1}GAP(X)))\otimes X.\end{eqnarray*}

In the formula, *Output* represents the features processed by the SE module, *σ*() stands for the Sigmoid activation function, while *δ*() denotes the ReLU activation function. *W*_1_ and *W*_2_ are the weights of the two fully connected layers. *GAP(X)* represents the global average pooling of input features *X*. Finally, ⊗ denotes channel-wise multiplication.

ECA module compresses spatial information using global average pooling. It then employs a lightweight 1D convolution to capture local cross-channel interactions. By generating channel attention weights with a sigmoid activation function, ECA performs an adaptive recalibration on the input features. This approach significantly improves model performance without a substantial increase in computational costs. The corresponding formula is as follows:

(9) \begin{eqnarray*}O{\mathrm{utput}}_{\mathrm{ECA}}=\sigma \left( {\mathbf{W}}_{k}\times G \right) \otimes X\end{eqnarray*}

where *W*_*k*_ is the 1D convolution kernel with a size of *k*, and *G* represents the channel descriptor after global average pooling.

In the iAMP-SeE model, we combined the SE and ECA mechanisms. This integration leverages the complementary advantages of both: the SE module’s global channel attention captures the overall structural features of the protein function, while the ECA module uses a lightweight convolution to retain local channel interactions and focus on key residue details. This design avoids the loss of fully connected information associated with SE and compensates for the limited receptive field of ECA, resulting in superior model performance compared to using either module in isolation.

### Hyperparameter tuning

The model employs a two-stage hyperparameter optimization strategy. Initially, a minimal functional model is constructed based on progressive scaling, where the validation loss is monitored while gradually increasing model complexity to preliminarily narrow down the parameter space. Subsequently, a grid search (Huang, Fu & Yu, 2025) is applied within the reduced hyperparameter range for precise tuning, systematically traversing all predefined hyperparameter combinations to identify the configuration that yields optimal model performance. The final optimized hyperparameters are detailed in Table 1.

### Oversampling technique

We primarily use oversampling techniques to address category imbalance. When certain categories have a significantly smaller number of samples, a model may become biased toward the majority class, which reduces its performance on the minority class. In this study, we used the SMOTE method to generate new samples by using linear interpolation within the feature space of the minority class samples. This method avoids simple duplication of samples and mitigates the risk of overfitting. The associated formula is as follows.

(10) \begin{eqnarray*}{x}_{\mathrm{new}}={x}_{i}+\lambda ({x}_{j}-{x}_{i}), \lambda \in [0,1].\end{eqnarray*}

In the aforementioned formula, *x*_*new*_ denotes the newly generated synthetic sample, *x*_*i*_ signifies a sample from the minority class, and *x*_*j*_ represents the nearest neighbor sample of *x*_*i*_. The difference between them corresponds to the vector difference in the feature space. *λ* denotes the interpolation coefficient, with a value range of [0,1].

**Table 1:** Hyperparameter tuning table.

**Category**	**Parameter name**	**Search range**	**Current value**
Feature extraction	esm_batch_size	[4,6,8,10,12,14,16]	8
CNN layer	conv1_filters	[64,128,256,512]	256
	conv1_kernel_size	[3,5,7,9]	5
	conv2_filters	[64,128,256]	128
	conv2_kernel_size	[3,5,7,9]	3
BiLSTM layer	lstm1_units	[128,256,512]	256
	lstm2_units	[128,256,512]	128
Attention mechanism	se_reduction_ratio	[4,6,8,10,12,14,16]	16
	eca_kernel_size	[3,7,9,11]	3
	cbam_kernel_size	[3,7,9,11]	7
Fully connected layer	dense1_units	[128,256,512,1024]	512
	dense2_units	[128,256,512]	256
	dense3_units	[64,128,256]	128
	dense4_units	[32,64,128]	64
	dropout_rate	[0.3,0.4,0.5,0.6,0.7]	0.5
	l2_regularization	[1e−5, 1e−4.1e−3]	1e−4
Training strategy	learning_rate	[1e−5.1e−4,1e−3]	1e−4
	batch_size	[8,16,32,64]	32
	epoch	[20,40,60,80,100,120,140,160,180,200]	120
	reduce_lr_factor	[0.2, 0.3,0.4,0.5,0.6,0.7,0.8]	0.5
	early_stopping_patience	[10,15,20,25,30]	20

### Cross validation

We used 10-fold cross-validation method (Ghobrial & Roth, 2025) to systematically evaluate the iAMP-SeE model’s performance on both binary and multi-class AMP classification tasks. The detailed design is as follows: first, the dataset is randomly and evenly divided into 10 mutually exclusive subsets. In each of the 10 experiments, nine subsets are used for training, while the remaining one is used for testing. This process is repeated 10 times to ensure that each subset is used as the test set exactly once. Finally, we calculate the average of the 10 validation results to get the final evaluation metric.

## Results and Discussion

### Binary classification performance comparison

**Figure 3:**
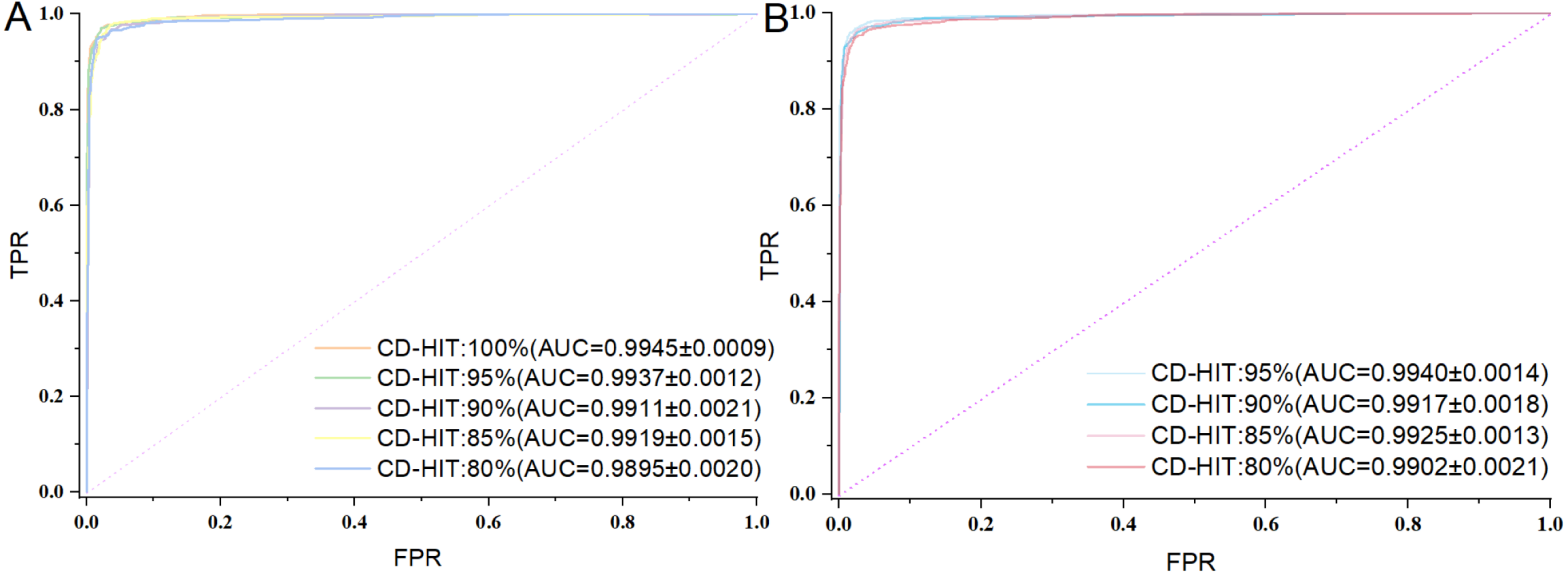
ROC curve comparison chart. (A) Balanced dataset; (B) imbalanced dataset.

For AMP binary classification, the iAMP-SeE model was initially trained and evaluated on Dataset 1 with a CD-HIT threshold of 100%. To assess the model’s robustness, the CD-HIT threshold was then progressively reduced by 5% increments until it reached 80%. At this stage, four datasets with imbalanced positive and negative samples were obtained. Each dataset was then used to train the model with iAMP-SeE, yielding the evaluation metrics of the model under different CD-HIT thresholds and imbalanced sample conditions, as shown in Table 2. Subsequently, based on the number of positive AMP sequences, negative samples were randomly selected to construct balanced datasets with equal numbers of positive and negative samples under different threshold conditions. Each dataset was used to train the model using iAMP-SeE, and the corresponding training results are presented in Table 3, while the ROC curves of the aforementioned model experiments are shown in Fig. 3.

**Table 2:** Comparative analysis of binary classification performance under class-imbalanced datasets. The quantities of AMPs and non-AMPs in each dataset are as follows: CD-HIT = 95%, AMPs (13,181); non-AMPs (15,949), CD-HIT = 90%; AMPs (10,038), non-AMPs (15,731); CDHIT = 85%: AMPs (8,899); non-AMPs (15,533); CD-HIT = 80%: AMPs (7,387), non-AMPs (15,297).

**CD-HIT**	**ACC**	**SEN**	**SPE**	**MCC**	**F1**
95%	0.9704 ± 0.0029	0.9651 ± 0.0054	0.9748 ± 0.0053	0.9403 ± 0.0058	0.9672 ± 0.0033
90%	0.9677 ± 0.0033	0.9627 ± 0.0052	0.9708 ± 0.0038	0.9321 ± 0.0072	0.9586 ± 0.0046
85%	0.9660 ± 0.0030	0.9588 ± 0.0101	0.9702 ± 0.0050	0.9269 ± 0.0064	0.9536 ± 0.0042
80%	0.9626 ± 0.0043	0.9546 ± 0.0080	0.9664 ± 0.0049	0.9155 ± 0.0095	0.9432 ± 0.0063

**Table 3:** Comparative analysis of binary classification performance under class-balanced datasets. The number of AMPs and non-AMPs in each dataset is as follows: CD-HIT = 95%, AMP (13,181); non-AMP (13,181); CD-HIT = 90%, AMP (10,038); non-AMP (10,038); CD-HIT = 85%, AMP (8,899), non-AMP (8,899); CD-HIT = 80%, AMP (7,387), non-AMP (7,387).

**CD-HIT**	**ACC**	**SEN**	**SPE**	**MCC**	**F1**
100%	0.9750 ± 0.0032	0.9741 ± 0.0037	0.9761 ± 0.0038	0.9479 ± 0.0049	0.9739 ± 0.0023
95%	0.9715 ± 0.0036	0.9680 ± 0.0050	0.9743 ± 0.0046	0.9424 ± 0.0073	0.9684 ± 0.0042
90%	0.9668 ± 0.0038	0.9593 ± 0.0109	0.9715 ± 0.0044	0.9302 ± 0.0082	0.9574 ± 0.0054
85%	0.9668 ± 0.0034	0.9546 ± 0.0096	0.9737 ± 0.0029	0.9282 ± 0.0074	0.9543 ± 0.0049
80%	0.9631 ± 0.0047	0.9497 ± 0.0094	0.9696 ± 0.0074	0.9165 ± 0.0098	0.9438 ± 0.0064

As can be seen from the aforementioned figures and tables, the iAMP-SeE model exhibits relatively stable recognition performance across a certain threshold range of the dataset. Furthermore, a comparison of the model results under varying positive-to-negative sample ratios demonstrates its robust performance, confirming the generalization capability of the iAMP-SeE model in AMP binary classification tasks.

To further validate the outstanding performance of the iAMP-SeE model in the binary classification task of AMPs, this study introduced several classic and novel binary classification recognition models for AMPs: sAMPpred_GAT (Ke et al., 2022), AMP-AttenPred (Xing et al., 2023), AMP-EBiLSTM, Deep-AMPEP30 (Yan et al., 2020), AMPEP (Bhadra et al., 2018), dsAMPGAN (Zhao et al., 2024), deep-AMPpred, and iAMPCN. All analyses utilized the dataset sequences described in Fig. 2A. Each model underwent the same number of training iterations, and 10-fold cross-validation was employed for performance comparison. The results are presented in Table 4.

**Table 4:** Comparative analysis of different AMPs binary classification models. Bold font indicates superior performance of the corresponding metric.

**Models**	**ACC**	**SEN**	**SPE**	**MCC**	**F1**	**AUC**
sAMPpred-GAT	0.7194 ± 0.1210	0.6722 ± 0.1686	0.7661 ± 0.3236	0.4740 ± 0.2641	0.7095 ± 0.0877	0.8173 ± 0.1556
iAMP-AttenPred	0.9550 ± 0.0049	0.9547 ± 0.0054	0.9553 ± 0.0080	0.9100 ± 0.0098	0.9549 ± 0.0052	0.9903 ± 0.0014
AMP-EBiLSTM	0.8611 ± 0.0051	0.8880 ± 0.0305	0.8341 ± 0.0336	0.7245 ± 0.0099	0.8647 ± 0.0054	0.9464 ± 0.0036
Deep-AMPEP30	0.9103 ± 0.0063	0.8969 ± 0.0078	0.9237 ± 0.0138	0.8210 ± 0.0128	0.9091 ± 0.0059	0.9632 ± 0.0034
AMPEP	0.8054 ± 0.0072	0.7526 ± 0.0156	0.8582 ± 0.0066	0.6143 ± 0.0136	0.7945 ± 0.0093	0.8813 ± 0.0055
dsAMPGAN	0.9715 ± 0.0036	0.9680 ± 0.0050	0.9743 ± 0.0046	0.9424 ± 0.0073	0.9684 ± 0.0042	0.9940 ± 0.0009
deep-AMPpred	0.9735 ± 0.0024	0.9718 ± 0.0039	0.9751 ± 0.0052	0.9472 ± 0.0063	0.9736 ± 0.0032	**0.9943 ± 0.0013**
iAMPCN	0.9675 ± 0.0029	0.9650 ± 0.0033	0.9716 ± 0.0043	0.9350 ± 0.0059	0.9673 ± 0.0030	0.9934 ± 0.0011
iAMP-SeE	**0.9750 ± 0.0032**	**0.9741 ± 0.0037**	**0.9761 ± 0.0038**	**0.9479 ± 0.0049**	**0.9739 ± 0.0023**	0.9942 ± 0.0012

As shown in Table 4, although iAMP-SeE’s AUC score is slightly lower than the deep-AMPpred model, it outperforms all other models across multiple performance indicators. This demonstrates the model’s excellent performance in AMP binary classification tasks.

### Multi classification performance comparison

#### Comparison before and after SMOTE oversampling

**Figure 4:**
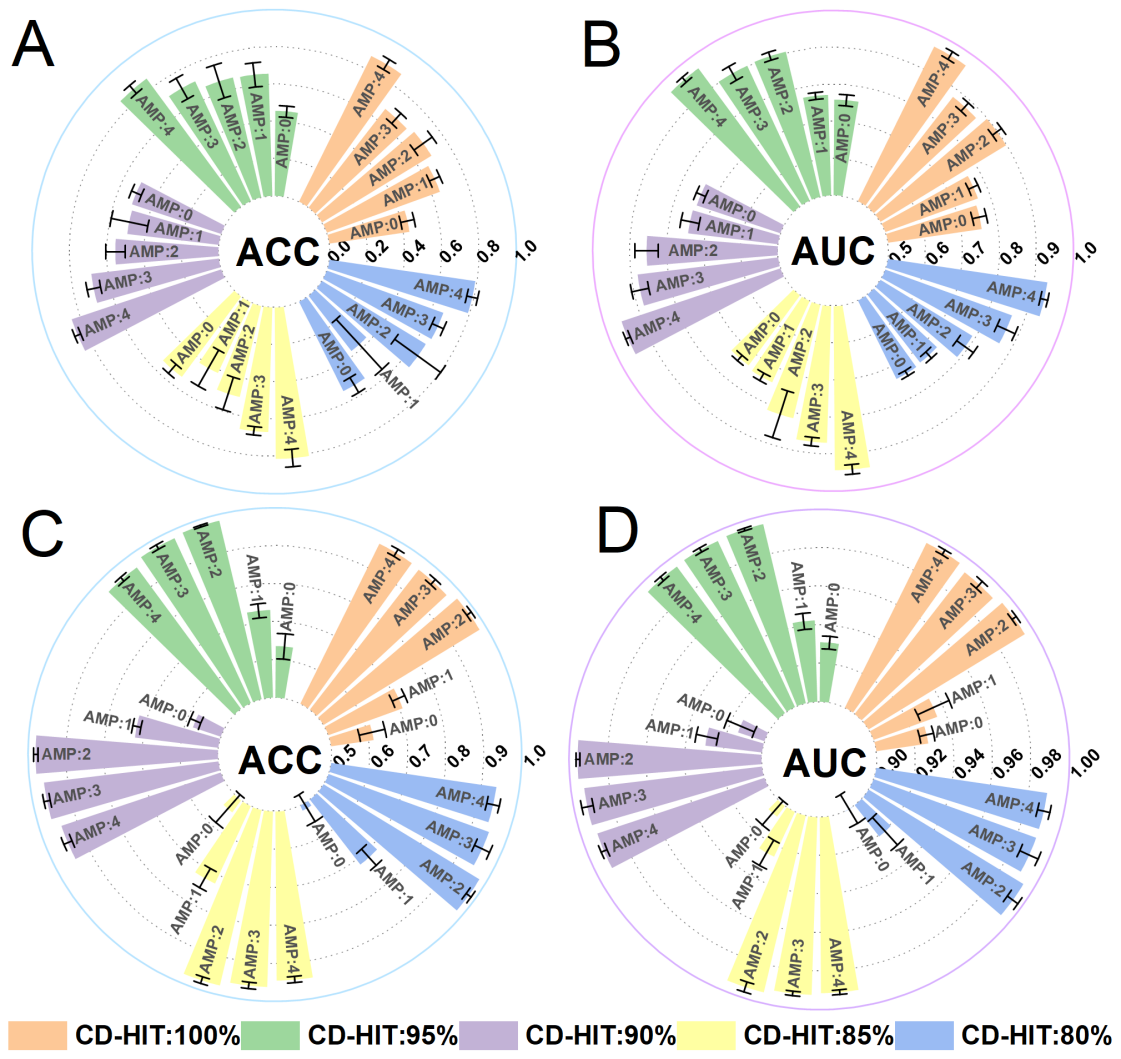
(A–D) Multiclass classification results under different CD-HIT thresholds. AMP0, Antibacterial; AMP1, Anti-Gram-positive; AMP2, Anti-Gram-negative; AMP3, Antifungal; AMP4, Antiviral; Subsequent charts/figures follow the same convention. (A/B), Model training results using raw data; C/D, Model training results using SMOTE-oversampled data.

In the multi-classification task, iAMP-SeE initially performs multi-classification recognition on the data from five positive sample categories in Dataset 1. It balances the quantitative differences between the data by employing the SMOTE oversampling technique. The oversampling method adopted in this study increases the number of all AMPs categories to match the largest single category through oversampling processing. Subsequently, the CD-HIT tool is utilized to reduce the percentage by 5% from 100% until the threshold reaches 80%. The model evaluation metrics for these five categories are compared before and after oversampling. To facilitate subsequent comparisons of model performance and other related factors, this study primarily evaluates the ACC and AUC model performance indicators in multi-classification tasks. The results are presented in Fig. 4.

Based on Fig. 4, it is evident that across various threshold ranges, the model’s ACC and AUC significantly surpass the original data after being oversampled using SMOTE, demonstrating that balancing data with SMOTE technology significantly enhances the model’s multi-classification capabilities. Additionally, it verifies that iAMP-SeE maintains a relatively stable model performance in identifying different AMPs across various thresholds. Specifically, it exhibits superior recognition and classification abilities for ‘Anti-Gram-negative’, ‘Antifungal’, and ‘Antiviral’ categories of AMPs. However, it is also noticeable that iAMP-SeE performs less effectively in identifying ‘Antibacterial’ and ‘Anti-Gram-positive’ categories compared to the other three categories of AMPs.

**Figure 5:**
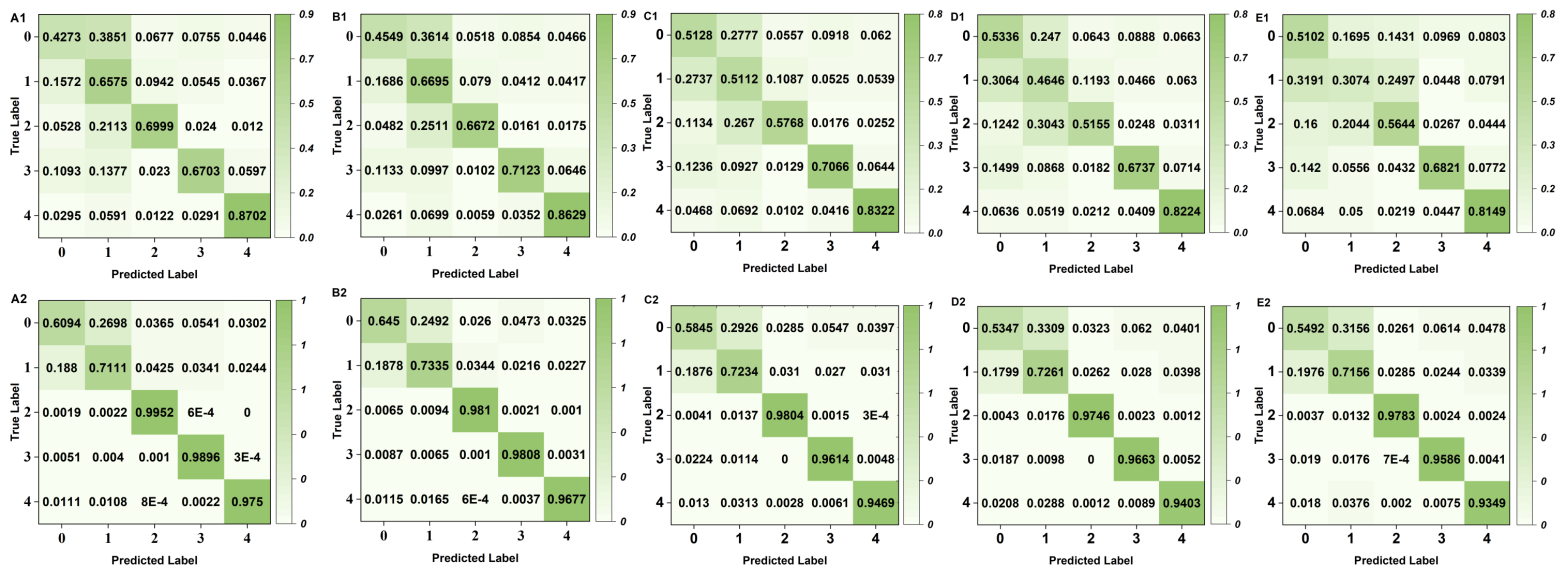
Confusion matrix comparison chart. CD-HIT = 100%, A1 raw data, A2 oversampled data; CD-HIT = 95%, B1 raw data, B2 oversampled data; CD-HIT = 90%, C1 raw data, C2 oversampled data; CD-HIT = 85%, D1 raw data, D2 oversampled data; CD-HIT = 80%, E1 raw data, E2 oversampled data.

To visually compare the model’s specific recognition abilities for each category before and after SMOTE oversampling, the confusion matrix (Leblebici, Çalhan & Cicioğlu, 2024) for the above five categories of iAMP-SeE at a CD-HIT threshold of 100%–80% is plotted, as illustrated in Fig. 5. Figure 5 clearly demonstrates the differences in the model’s recognition abilities for each category before and after SMOTE oversampling, rigorously verifying that balancing data with SMOTE can enhance the model’s multi-classification capabilities for AMPs.

#### Model comparison

To preliminarily validate the recognition capability of the iAMP-SeE model in multi-classification tasks, this study compares it with iAMP-CA2L (Xiao et al., 2021), TransImbAMP (Pang et al., 2022), deep-AMPpred and iAMPCN using the aforementioned multi-category dataset with a CD-HIT threshold of 100%. The same number of model training iterations and 10-fold cross-validation were also employed for model evaluation. The comparison results are presented in Table 5.

**Table 5:** Comparative analysis of different AMPs multiclass classification models. Bold font indicates superior performance of the corresponding metric.

**Method**	**Performance metrics**	**0**	**1**	**2**	**3**	**4**
iAMP-CA2L	ACC	0.4840 ± 0.0638	0.5993 ± 0.0729	0.8198 ± 0.0468	0.8521 ± 0.0424	0.8854 ± 0.0470
	ROC AUC	0.8801 ± 0.0164	0.9212 ± 0.0172	0.9701 ± 0.0121	0.9797 ± 0.0090	0.9840 ± 0.0063
TransImbAMP	ACC	0.4671 ± 0.0320	0.6502 ± 0.0573	0.8394 ± 0.0596	0.9099± .0534	0.8672 ± 0.0496
	ROC AUC	0.8521 ± 0.0209	0.9434 ± 0.0138	0.9784 ± 0.0068	0.9873 ± 0.0047	0.9817 ± 0.0098
deep-AMPpred	ACC	**0.6359 ± 0.0334**	0.7009 ± 0.0185	0.9789 ± 0.0025	0.9582 ± 0.0093	0.9625 ± 0.0065
	ROC AUC	**0.9309 ± 0.0033**	**0.9422 ± 0.0042**	0.9927 ± 0.0016	0.9891 ± 0.0032	0.9945 ± 0.0013
iAMPCN	ACC	0.5047 ± 0.0287	0.6540 ± 0.0297	0.8533 ± 0.0515	0.8887 ± 0.0371	0.9462 ± 0.0172
	ROC AUC	0.7385 ± 0.0134	0.8445 ± 0.0146	0.8543 ± 0.0314	0.8823 ± 0.0238	0.9526 ± 0.0070
iAMP-SeE	ACC	0.6128 ± 0.0345	**0.7077 ± 0.0180**	**0.9813 ± 0.0071**	**0.9667 ± 0.0092**	**0.9696 ± 0.0106**
	ROC AUC	0.9272 ± 0.0037	0.9351 ± 0.0090	**0.9951 ± 0.0014**	**0.9931 ± 0.0024**	**0.9955 ± 0.0015**

It can be seen from Table 5 that iAMP-SeE is superior to other models in the categories of ‘Anti-Gram-negative’, ‘Antifungal’, and ‘Antiviral’, but it still has no advantage in identifying ’Antibacterial’ and ‘Anti-Gram-positive’ categories, which indicates that iAMP-SeE has certain differences in identifying various types of AMPs. Analysis of the confusion matrix in Fig. 5 reveals a high degree of overlap between the ‘Antibacterial’ and ‘Anti-Gram-positive’ categories during model prediction. From a biological perspective, this occurs because “Antibacterial” is a broader concept that encompasses a certain number of “Anti-Gram-positive” peptides. Although identical AMP sequences were removed using the CD-HIT tool, a proportion of sequences still exhibit similarity, leading to confusion in the classification of these two types of AMPs and consequently limiting the model’s discriminative performance.

To further validate the performance of the iAMP-SeE model, this study introduced Dataset 2, which was derived from the multi-class AMP classification task in deep-AMPpred and processed with CD-HIT at 40% sequence identity to remove redundancy. Using this dataset, the model was trained for multi-class AMP classification to evaluate the detailed categorization capability of iAMP-SeE. The corresponding results are presented in Table 6.

**Table 6:** Multiclass classification results for Dataset 2.

**Activity**	**ACC**	**AUC**
antibacterial	0.6805 ± 0.0476	0.9430 ± 0.0094
antibiofilm	0.8018 ± 0.0529	0.9735 ± 0.0047
anticancer	0.9204 ± 0.0266	0.9858 ± 0.0076
antifungal	0.8867 ± 0.0370	0.9864 ± 0.0061
antigram_neg	0.7150 ± 0.0206	0.9651 ± 0.0098
antigram_pos	0.7921 ± 0.0464	0.9721 ± 0.0084
antimicrobial	0.5177 ± 0.0409	0.8705 ± 0.0241
antiviral	0.8766 ± 0.0416	0.9853 ± 0.0079
anti_mammalian_cells	0.9240 ± 0.0385	0.9888 ± 0.0059

A comparison with data from Zhao et al. (2024) (Table S1) confirms the excellent performance of the iAMP-SeE model in the multi-class AMPs classification task. Although it does not achieve the highest accuracy in a minority of AMPs categories, the model exhibits superior performance in the majority of classes. Notably, iAMP-SeE shows a comprehensively leading performance in terms of the AUC metric, confirming its advanced capability in the multi-class identification of AMPs.

### Ablation study

Ablation experiments are a standard research method in machine learning and natural language processing (Zhang et al., 2024). They help analyze how each component of a model contributes to its overall performance. In this study, we performed ablation experiments for both binary and multi-class classification tasks by replacing the feature extraction method and various modules within the iAMP-SeE model.

**Figure 6:**
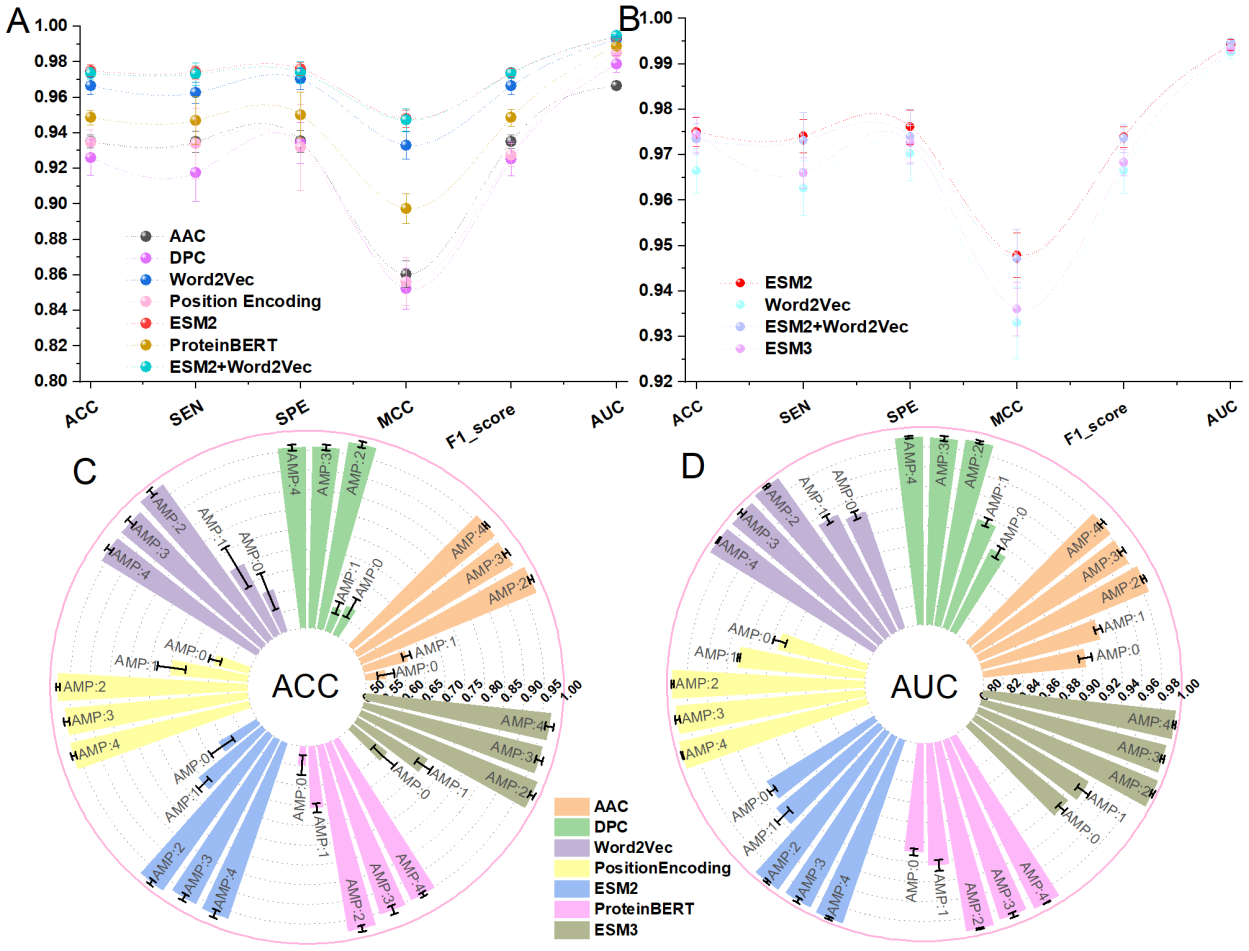
Comparative analysis of feature extraction methods. (A & B), Binary classification results for AMPs; (C & D), Multiclass classification results for AMPs.

In the ablation experiment of this study, the data utilized came from dataset 1 with a CD-HIT threshold of 100%. The selection of feature extraction methods employed in the study is illustrated in Fig. 6. Upon comparing the evaluation metrics of binary and multi-class models across various models, it is evident from Fig. 6 that the model evaluation metrics obtained by training ESM2 on the same model are in the leading position. To investigate whether the more complex and innovative ESM3 feature extraction (Shao & Liu, 2025) method can further enhance the model’s relevant performance, it was introduced into various classification tasks for model performance comparison. As shown in Fig. 6, although ESM3 achieved relatively excellent results, it did not completely surpass ESM2. This situation may arise due to ESM3’s reduced sensitivity to short data caused by its large model size when handling short sequence tasks. The ablation experiment of the feature extraction method demonstrates that ESM2 can capture relevant information in protein sequences more efficiently.

**Figure 7:**
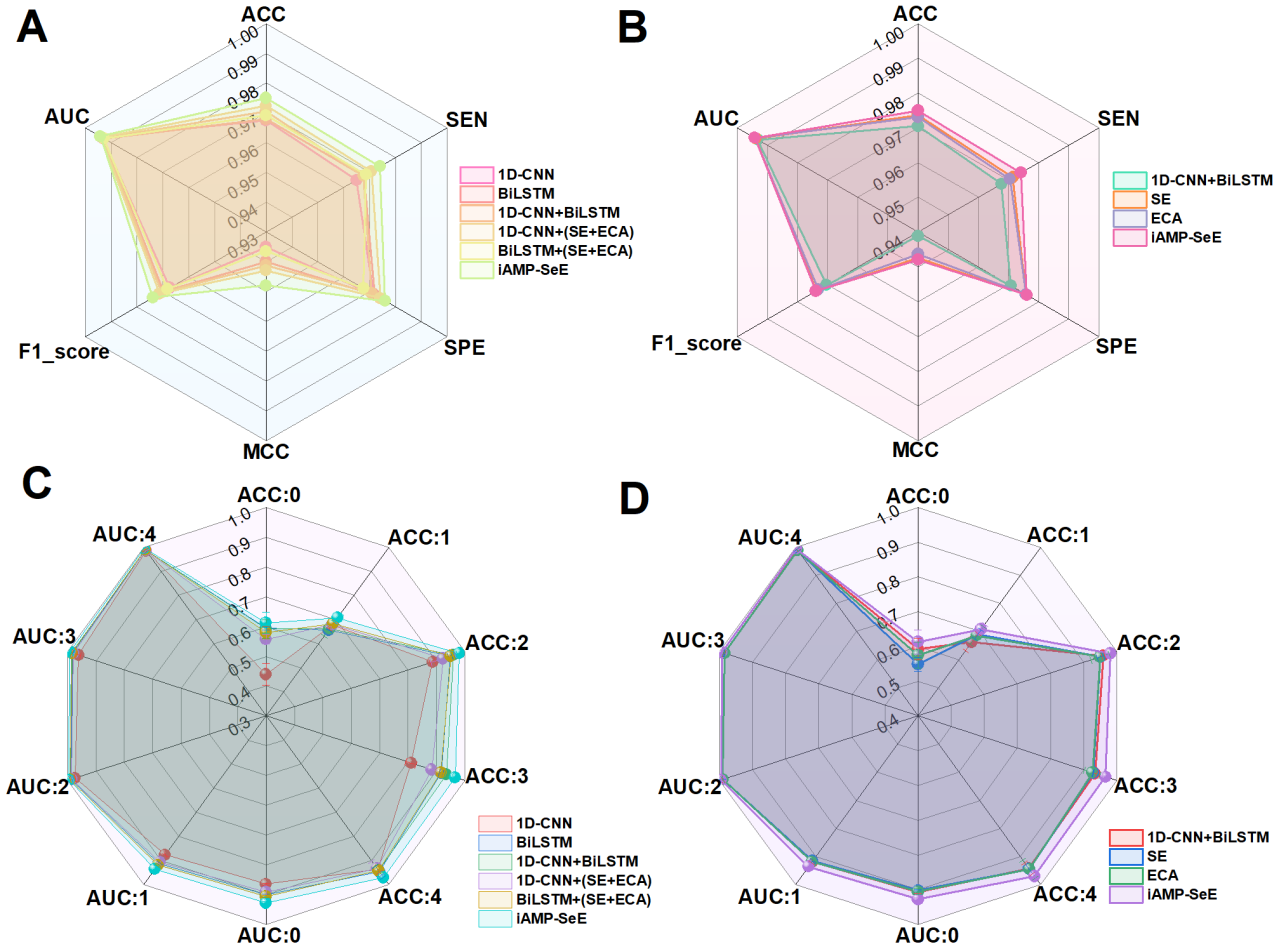
(A–D) Ablation studies across modules.

In the follow-up ablation experiment, the relevant evaluation indexes of the model were compared by adding or subtracting the modules of the relevant model. The specific experimental scheme is: the combination of 1D-CNN, BiLSTM, SE and ECA modules. The results are shown in Fig. 7.

Figures 7A and 7C show that iAMP-SeE achieves the best performance. The performance of the other models declines because they lack the additional modules. This indicates that the complementary feature extraction capabilities of CNN, BiLSTM, and SE+ECA modules, coupled with the dynamic optimization of the attention mechanism, can enhance model performance to a certain extent. Similarly, as can be seen in Figs. 7B and 7D, the attention mechanisms of SE, and ECA can enhance the performance of AMPs binary and multiclass 1D-CNN+BiLSTM models. To verify whether combining attention mechanisms can leverage the strengths of different modules and overcome the limitations of individual attention mechanisms, this study integrates the aforementioned two attention mechanisms into the 1D-CNN+BiLSTM model. Through experimental comparison, it has been found that the module, formed by the combination of SE+ECA, achieves the best evaluation metrics across all models in binary classification, and also performs optimally in most categories within multi-classification tasks. Ablation studies were performed to validate the effectiveness of the combination strategy among the modules in iAMP-SeE.

### Model interpretability analysis

Although iAMP-SeE demonstrates promising performance in AMPs classification tasks, it remains crucial to investigate whether the model can accurately capture biologically relevant information. To this end, we conducted multi-faceted interpretability analyses.

**Figure 8:**
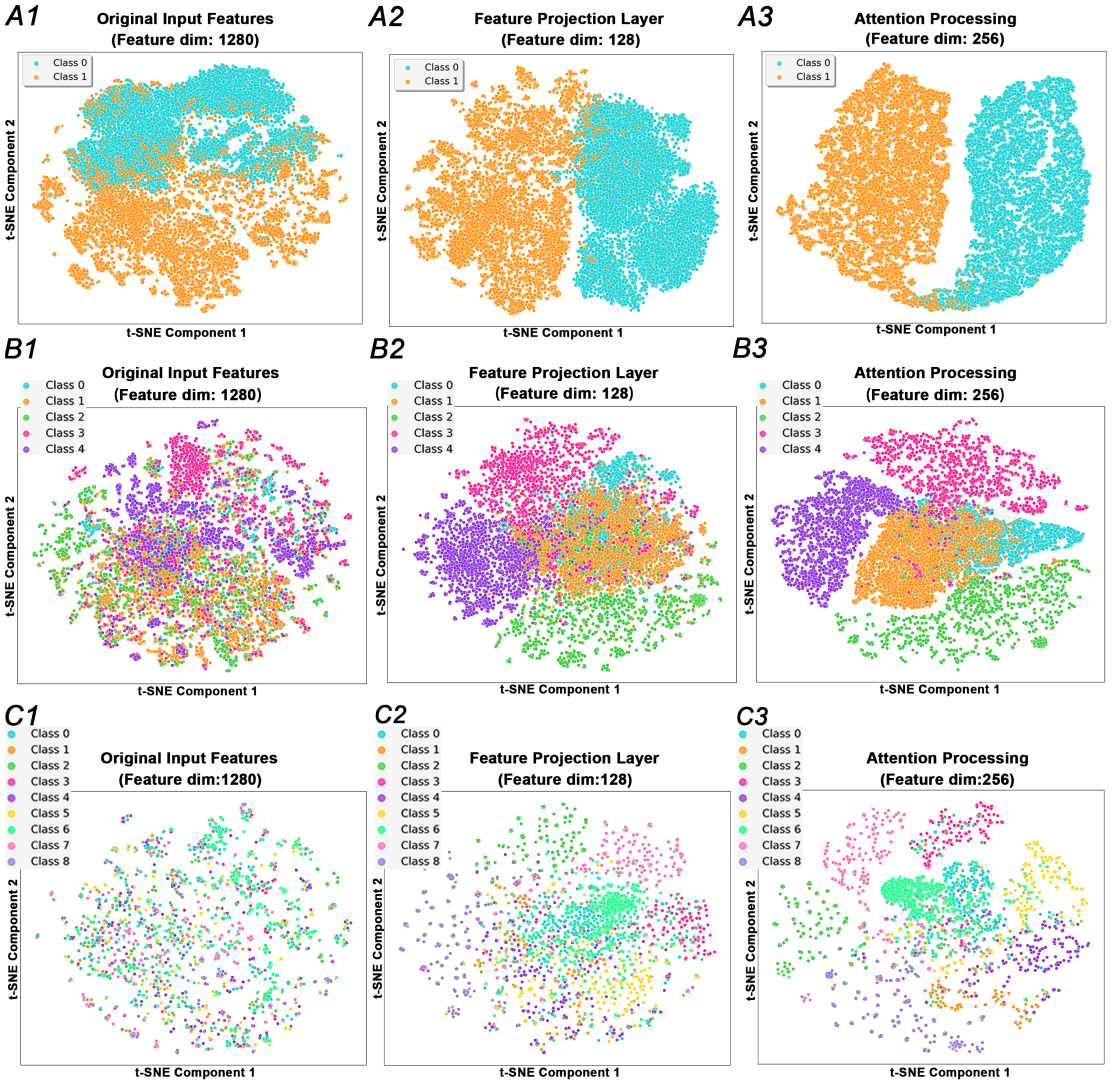
T-SNE analysis plots. Panels (A) and (B) illustrate the t-SNE visualizations for binary and multi-class classification of AMPs using the self-constructed dataset (CD-HIT = 100%). Panel (C) presents the t-SNE visualization for multi-class classification of AMPs using the publicly available dataset from Zhao et al. (CD-HIT = 40%).

First, t-SNE (t-distributed Stochastic Neighbor Embedding) (Maaten & Hinton, 2008) was employed to visualize the feature space of the dataset samples encoded by ESM2. By mapping high-dimensional features onto a 2D plane, t-SNE enables an intuitive display of the distribution of different categories in the feature space. Using the 1280-dimensional embeddings from ESM2 as the initial input source, we compared the feature distributions of the iAMP-SeE model at three stages: original input features, features after feature projection layer, and features processed by the combined SE and ECA attention mechanisms. The distributions are shown in Fig. 8. By comparing the t-SNE visualization results across the three stages in Fig. 8, it can be observed that the iAMP-SeE model successfully transforms the original high-dimensional ESM-2 features into task-specific discriminative features, enabling effective classification of complex samples that originally overlapped significantly and were difficult to distinguish directly. Notably, the SE and ECA attention mechanisms, by re-weighting important channels and spatial features, enhance intra-class compactness and increase inter-class separation, ultimately yielding clear clustering patterns. This also demonstrates the strong interpretability and outstanding classification effectiveness of the iAMP-SeE model in mining deep functional features of AMP protein sequences.

**Figure 9:**
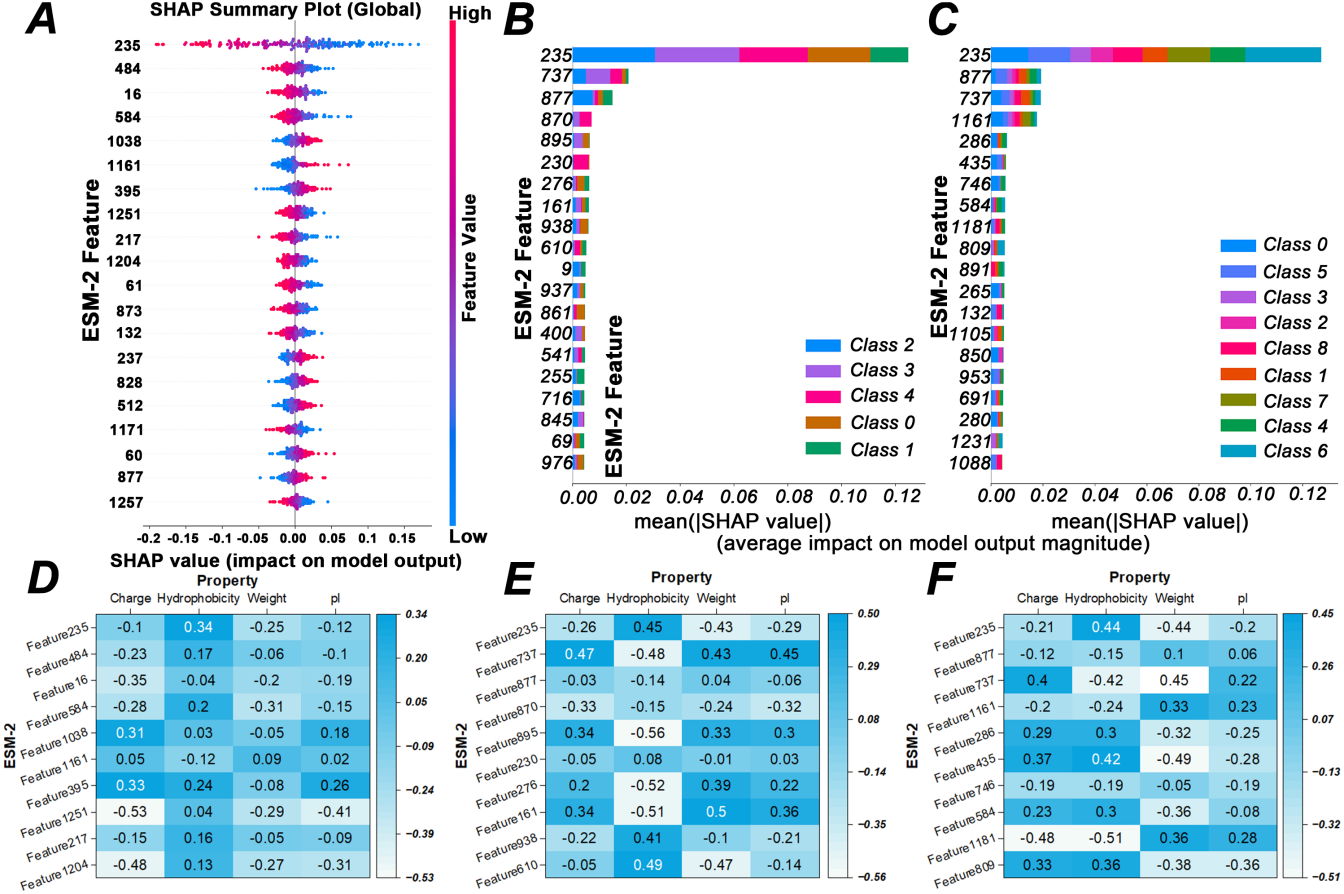
Interpretability analysis of deep learning model *via* ESM-2 feature evolution and biological correlation. Panels (A) and (B) present SHAP contribution analysis plots for binary and multi-class AMPs classification using a self-constructed dataset. Panel (C) displays a SHAP contribution analysis plot for multi-class AMPs classification employing the publicly available dataset from Zhao et al. (2024). Panels (D, E), and (F) illustrate comprehensive analysis plots for Spearman correlation with physicochemical properties. “Charge” refers to the net charge of the sequence at physiological pH, “Hydrophobicity” represents the tendency of amino acid residues to avoid water molecules, “Weight (Molecular Weight)” denotes the sum of the masses of all amino acid residues in the sequence, and “pI (Isoelectric Point)” indicates the pH at which the net charge of a protein is zero.

To further understand which key features play critical roles in the model’s recognition and classification, we employed SHapley Additive exPlanations (SHAP) (Lundberg & Lee, 2017) analysis to quantify the contribution of each feature to the model’s predictions, thereby providing a deeper explanation of the protein sequence classification results. The results are shown in Figs. 9A–9C, which respectively display the top 20 ESM2 feature dimensions contributing most to the model’s decisions for each AMP recognition task. It can be clearly observed that ‘ESM2 Feature 235’ has the highest contribution across all classification tasks, demonstrating its pivotal role in the model’s ability to classify AMPs.

To further investigate these decisive features and reveal the intrinsic logic underlying the model’s discrimination of AMPs, we focused on the distinctive structural characteristics of antimicrobial peptides: the cationic nature due to an abundance of positively charged residues such as arginine and lysine, and the typical amphipathic structure containing both hydrophilic and hydrophobic regions (Laguera et al., 2025). By correlating the ESM2 feature vectors with key physicochemical properties: average hydrophobicity and normalized net charge, we performed Spearman correlation analysis (De Winter, Gosling & Potter, 2016) between the top 10 ESM2 features that contribute most to the model’s decisions and the biophysical properties of the proteins. This was conducted to assess whether monotonic relationships exist between the abstract high-dimensional features extracted by ESM2 and known biophysical properties. The results are presented in Figs. 9D–9F. From these, we observe that the model does not learn randomly; instead, it accurately captures feature patterns highly associated with biological activity. In particular, the key feature ‘ESM2 Feature 235’ shows a significant correlation with hydrophobicity, indicating that this feature is actually used by the model to capture typical biological characteristics of AMPs.

The interpretability analysis fully validates the credibility and rationality of the iAMP-SeE model in the task of AMPs classification and recognition. This mapping from the mathematical high-dimensional space to fundamental physicochemical properties not only demonstrates that the decisions of the iAMP-SeE model are not blind guesses, but also reveals that ’ESM2 Feature 235’ serves as a crucial bridge connecting the black box of deep learning with the functional essence of AMP proteins.

## Conclusions

To enhance the model’s ability to recognize AMPs, this study developed an iAMP-SeE model suitable for both binary and multi-class classification of AMPs. Utilizing the ESM2 protein feature extraction method, the model efficiently captures the sequence information of AMPs within the dataset. Additionally, the model incorporates the high-performing SE+ECA module into a multi-layer structure composed of CNN and BiLSTM. Through the synergy of these three modules and the complementarity of the SE and ECA attention mechanisms, the model achieves the recognition of AMPs-related sequence information features and their corresponding classification. By comparing the performance metrics of iAMP-SeE across various CD-HIT thresholds and against existing AMPs recognition models, it is evident that this model outperforms others in most evaluation metrics for binary classification tasks. In multi-class AMPs recognition tasks, SMOTE oversampling is employed to address category imbalance. Experimental comparisons demonstrate that iAMP-SeE excels in multi-class AMPs recognition tasks. Furthermore, rigorous ablation studies reveal the model’s performance under the collaborative efforts of its various modules, confirming its superior performance in AMPs recognition. The relevant interpretability analysis of the iAMP-SeE model further reduces its black-box nature and enhances its trustworthiness. Consequently, it can be concluded that iAMP-SeE offers valuable insights for both binary and multi-class AMPs recognition tasks.

Although the iAMP-SeE model proposed in this study has achieved relatively good results in AMPs recognition, it still has shortcomings. Firstly, the number of AMPs and data categories used in the study are limited. In future work, the performance of AMPs models in other different categories should be further tested. Secondly, iAMP-SeE failed to achieve the best results in some AMPs categories, indicating that the model still lacks recognition ability in these categories. Therefore, the model should be further optimized for AMPs of these categories in the future.

##  Supplemental Information

10.7717/peerj.20978/supp-1 Supplemental Information 1 Data and code

10.7717/peerj.20978/supp-2 Supplemental Information 2 README

10.7717/peerj.20978/supp-3 Supplemental Information 3 Uses the iAMP-SeE model to perform binary classification for identifying AMPs

10.7717/peerj.20978/supp-4 Supplemental Information 4 Uses the iAMP-SeE model to perform multi-label classification for identifying AMPs

10.7717/peerj.20978/supp-5 Supplemental Information 5 Multiclass classification results for dataset 2 Data sourced from Zhao et al.

## Additional Information and Declarations

### Data Availability

The following information was supplied regarding data availability: The source code is publicly available at GitHub and Zenodo: – https://github.com/cqw0715/iAMP-SeE.git
– Chen, Q. (2025). iAMP-SeE: An Antimicrobial Peptide Recognition Model Based on ESM2 Feature Extraction and Hybrid Attention Mechanisms [Data set]. Zenodo. https://doi.org/10.5281/zenodo.17398951. The deep-AMPpred dataset is available at GitHub:
https://github.com/JunZhao-hash/deep_AMPpred.
