# Supplementary material for: iAMP-SeE: an antimicrobial peptide recognition model based on ESM2 feature extraction and hybrid attention mechanisms
Source: PeerJ. 2026 Mar 26;14:e20978. doi: 10.7717/peerj.20978 (PMC13033287; doi:10.7717/peerj.20978)
Supplement: Supplemental Information 5 — Data sourced from Zhao et al. [file peerj-14-20978-s005.docx]

| **Activity** | **Method** | **ACC** | **AUC** |
| --- | --- | --- | --- |
| antibacterial | iAMP-CA2L | 0.4987 | 0.4879 |
|  | iAMPCN | 0.8130 | 0.8122 |
|  | deep-AMPpred | 0.7361 | 0.8126 |
| antibiofilm | iAMP-CA2L | 0.5338 | 0.4786 |
|  | iAMPCN | 0.7176 | 0.8263 |
|  | deep-AMPpred | 0.9887 | 0.8344 |
| anticancer | iAMP-CA2L | 0.7149 | 0.4933 |
|  | TransImbAMP | 0.4261 | 0.3727 |
|  | iAMPCN | 0.7003 | 0.7828 |
|  | deep-AMPpred | 0.8369 | 0.7854 |
| antifungal | iAMP-CA2L | 0.5000 | 0.4982 |
|  | TransImbAMP | 0.5731 | 0.5302 |
|  | iAMPCN | 0.8291 | 0.8248 |
|  | deep-AMPpred | 0.8318 | 0.8345 |
| antigram_neg | TransImbAMP | 0.4203 | 0.4088 |
|  | iAMPCN | 0.7131 | 0.7768 |
|  | deep-AMPpred | 0.7323 | 0.7923 |
| antigram_pos | TransImbAMP | 0.5439 | 0.4398 |
|  | iAMPCN | 0.7353 | 0.7848 |
|  | deep-AMPpred | 0.7421 | 0.7907 |
| antiviral | iAMP-CA2L | 0.4980 | 0.5258 |
|  | TransImbAMP | 0.4689 | 0.3518 |
|  | iAMPCN | 0.7886 | 0.8443 |
|  | deep-AMPpred | 0.8607 | 0.8696 |
| anti_mammalian_cells | TransImbAMP | 0.4776 | 0.4797 |
|  | iAMPCN | 0.7479 | 0.8208 |
|  | deep-AMPpred | 0.8378 | 0.8075 |
